# Superior Vena Cava Syndrome and Wallstent: A Systematic Review

**DOI:** 10.3400/avd.ra.21-00118

**Published:** 2022-06-25

**Authors:** Ali Kordzadeh, Alan Askari, Muhammad A. Hanif, Vijay Gadhvi

**Affiliations:** 1 Mid & South Essex Hospital Foundation Trust, Basildon, Essex, UK; 2 Cambridge University Hospitals NHS Foundation Trust, Cambridge, UK; 3 Anglia Ruskin University, Faculty of Health, Education, Medicine and Social Sciences, Cambridge, UK

**Keywords:** superior vena cava (SVC), superior vena cava syndrome, malignancy, endovascular therapy (ET), Wallstent, systematic review

## Abstract

**Purpose:** To elucidate the indication, presentation, demographics, Stanford classification, technical efficacy, morbidity, mortality and long term patency of Wallstent for superior vena cava (SVC) syndrome.

**Materials and Methods:** A systematic review of literature in Pubmed and Embase, CINAHL and Cochrane Library in accordance to PRIMSA was conducted. Retrieval and extraction was performed by two independent reviewers with inter-rater reliability test. The hierarchy of the evidence was assessed through the National Institute for Health and Care Excellence Checklist. Data was subjected to pooled prevalence analysis, Cox regression, Kaplan–Meir survival and test of probability using log rank analytics. This review is registered with International prospective register of systematic review: CRD42021271009.

**Results:** A total of n=701 individuals with n=930 stents with median age of 60 (interquartile range (IQR): 26–89) years and male predominance 3.5 : 1 were identified in n=30 articles. The most common venographic classification was Stanford type II (n=344, 50%) and complete symptomatic resolution was achieved in 48 h. The 30-day morbidity was (n=62, 8%) and mortality was (n=21, 3%). Female gender was associated with higher 30-day morbidity (p<0.03). The cumulative median patency of Wallstent for non-malignant aetiology was [550 days (IQR: 14–1080) vs. 120 days (IQR: 0–925)] for malignancy (p<0.03).

**Conclusion:** The use of Wallstent for resolution of malignancy induced SVC syndrome as a first line therapy is feasible and associated with low mortality. Their use for non-malignant aetiology demands a more in depth review and advocates further investigation.

## Introduction

Superior vena cava (SVC) syndrome refers to a groups of symptoms such as oedema (facial and arms), shortness of breath, conjunctival suffusion, coughing and stridor as a consequence of partial or complete SVC obstruction. In some series, sever neurological symptoms (stupor and coma) or airway compromise has also been reported.^1)^ Their reported incidence ranges from 1 in 650–3100 cases and in USA alone 15,000 cases are reported annually. The first report of SVC syndrome dates back to 1757 when William Hunter described an extrinsic compression of the SVC by a large aneurysm secondary to syphills.^2)^ In order of prevalence, mediastinal malignancy (bronchogenic, lymphoma, metastatic) remain the most common aetiology (70%) followed by infectious and intragenic (Indwelling access and pacemakers) injuries.^3)^ The treatment aims at reduction of the venous pressure either by medical management or surgery (open or endovascular). Open repair using prosthetic (polytetrafluoroethylene [PTFE] or Dacron) or autogenous vein (spiral saphenous vein or femoral vein) is now reserved if endovascular approach fails to prevail as later is associated with lower morbidity and mortality.^4,5)^ Since early 1990s, Wallstent endo-prosthesis (self-expanding stent) has been routinely deployed for tackling the SVC syndrome amongst other stents such as Z stent. However, to date no systematic review has evaluated the independent outcome of Wallstents on their long-term technical efficacy, associated mortality and morbidity in the literature. In addition, there is no clear consensus or guidelines for their use that was originally designed for other purpose. We routinely use Wallstent in our unit and we could not suggest any long term outcomes to our patients due to lack of robust evidence. Therefore, the aim of this systematic review to is to establish the indication, classification, technical efficacy, morbidity, mortality and longevity of Wallstent for the treatment of SVC syndrome.

## Materials and Methods

### Search strategy

A systematic review of literature from the database inception to 1st of August 2021 in Pubmed, Embase, CINAHL and Cochrane Library in accordance to the Preferred Reporting Items for Systematic reviews and Meta-Analysis (PRIMSA) was conducted.^6)^ Medical Subject Headings (MeSH) terms or keywords included: “venae cavae” [MeSH Terms] OR vena cava [Text Word], Wallstent [MeSH Terms] OR Wallstent [text Word]. References of the retrieved articles were also manually evaluated for any additional literature not identified in the initial search. All abstracts were retrieved and reviewed by two separate investigators. Studies that appeared to fulfil the eligibility criteria but had an insufficient information in the abstracts were also retrieved and examined in full. The data extraction was also performed by two separate investigators and inter-rater reliability [Cohen Kappa Coefficient was (***k***)] was calculated. This systematic review was also registered with International Prospective Register of Systematic Review (PROSPERO) National Institute for Health Research, UK with registration (NIHR) number: CRD42021271009.

### Selection criteria

All studies involving humans, pertained to the use of Wallstent for SVC syndrome for any given aetiology in English language were selected for inclusion. Published material that were experimental studies, narrative reviews and expert opinion were excluded.

### Statistical analysis

To achieve an informed conclusion and evidence-based approach, the included articles were evaluated for their validity, bias, applicability and inference using critical appraisal tool provided by Oxford Critical Appraisal Skills programme (CASP). Due to a lack of consistency of data and its randomisation, a meta-analysis was not feasible. However, a pooled analysis was conducted by calculation of the median value along with their interquartile range (IQR). The data output was calculated and presented with percentile of each category. Sub-group analysis was performed using Cox regression to evaluate the impact of various attributes (age, gender, Stanford classification [Type I–IV]) over a median time on the endpoint of 30-day mortality and morbidity (binary) outcomes. In addition, a Kaplan–Meir survival analysis was used to see the difference between the survival (long-term patency) of Wallstent in malignant versus non-malignant aetiology with log rank test of probability (p-value).

### Venographic classification

SVC syndrome is classified into four different subgroups according to Stanford. This venographic classification is primarily based on degree of obstruction, valve competency and collateral venous flow (intercostal, left accessory hemi-azygous, azygous, hemi-azygous, para-vertebral and internal mammary veins). Type I, is a partial stenosis up to 90% of the SVC with patent Azygous vein. Type II is near total occlusion (90%–100%) of SVC with flow from azygous vein to the right atrium. Type III is complete occlusion of SVC with reverse flow in azygous vein and finally, Type IV is complete obstruction of SVC with one or more than one collateral vein occlusion^7)^ (**Table 1**).

**Table 1:** Table 1 Venographic and symptom classification of SVC syndrome

Type	Venographic features
Stanford I	Partial stenosis up to 90% of the SVC with patent azygous vein
Stanford II	Near total occlusion (90%–100%) of SVC with flow from azygous vein to the right atrium
Stanford III	Complete occlusion of SVC with reverse flow in azygous vein
Stanford IV	Complete obstruction of SVC with one or more than one collateral vein occlusion
Grade	Kishi symptomatic classification
Grade I	Any signs of venous congestion
Grade II	Nasal or fascial oedema
Grade III	Laryngeal oedema
Grade IV	Central nerves related symptoms

SVC: superior vena cava

### Symptomatic classification

Symptomatology of SVC syndrome has been classified into four grades by Kishi et al. in 1993. According to this (a simplistic version) a grade I relates to any signs of venous congestion, grade II refers to nasal or fascial oedema, grade III refers to laryngeal oedema and Grade IV is central nerves related symptoms. In this categorisation grade IV represents the most severe presentation that requires urgent assessment and management as delay in treatment could result in fatality^8)^ (**Table 1**).

### Definitions

Technical success within this review was defined as post endovascular venography evidence of patency with resolution of symptoms. Primary patency was defined as patency of the Wallstent endo-prosthesis requiring no further assistance for its luminal patency and symptomatic relief. Secondary patency was defined as any intervention (venoplasty, stent extension, thrombolysis, open surgery) to keep the original Wallstent prosthesis open following its primacy patency. Mortality was defined as death from the Wallstent endo-prosthesis placement within 30 days and complications as any event that arose from the procedure requiring further intervention.

## Results

**Figure 1:**
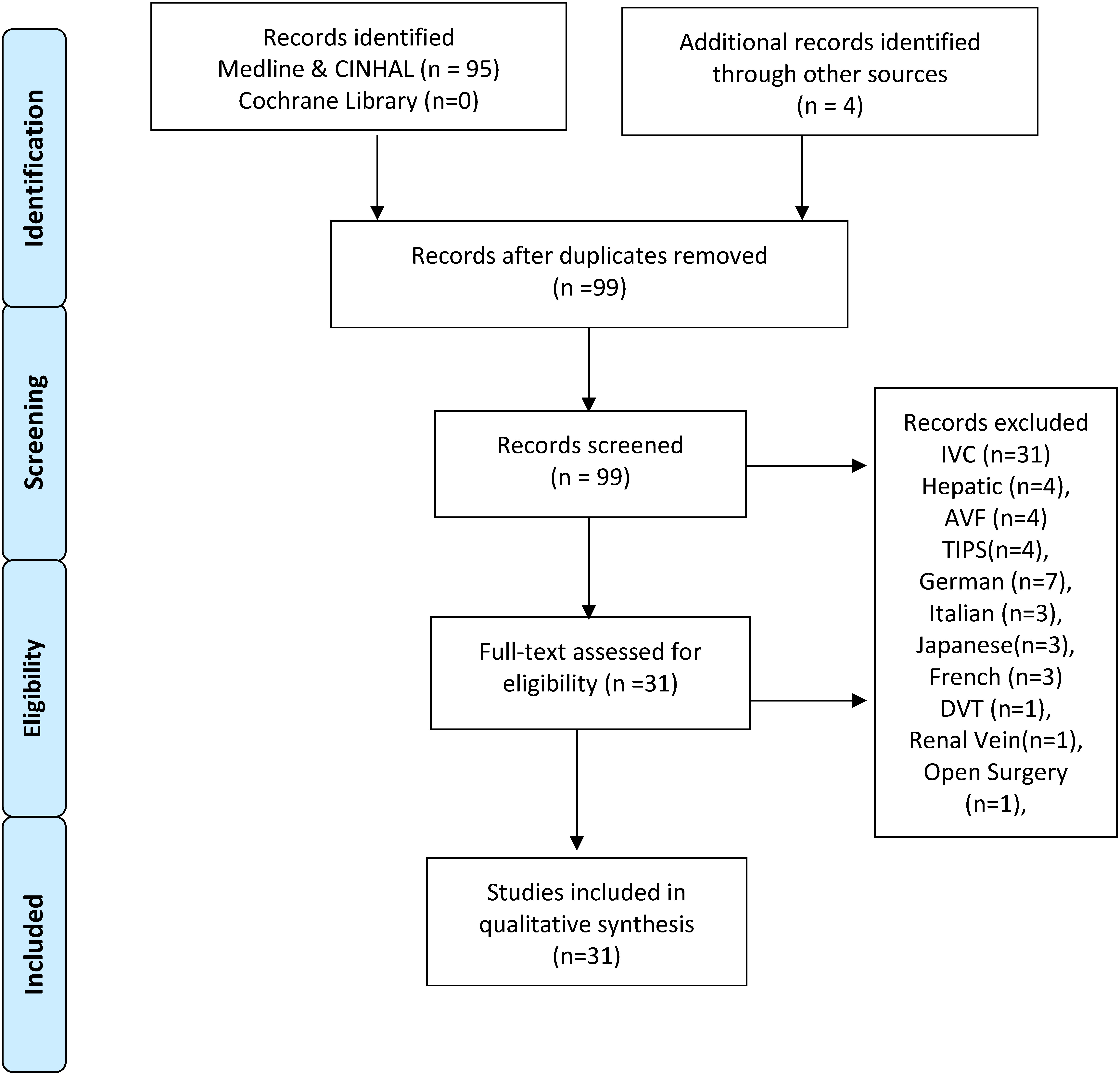
Fig. 1 Preferred reporting items for systematic reviews and meta-analysis flow-chart.

Total of n=99 articles were identified with no systematic or Cochrane review in the literature. All articles were found to be of case reports or cohort (grade and level of evidence: class III/ IIb, level C/D). The overall missing data was 2.5% (indication, gender, classification and follow up).^9–11)^ After application of the inclusion criteria, total of n=30 articles was eligible. The PRISMA flow chart is highlighted in **Fig. 1**. Inter-rater reliability was 0.88 for study retrieval and 0.86 for data extraction.

A total of n=701 individuals with n=930 stents were identified of the n=31 articles. The aetiology for SVC syndrome was n=643 (92%) for malignancy and rest for non-malignant condition (pacemaker, indwelling central lines, fibrosis) n=36 (6%). There was a male predominance 3.5 : 1 [male n=543 (78%) vs. female n=152 (22%)]. The median age of the group was 60 years (IQR: 26–89 years). The most common venographic classification in the order of prevalence was Stanford type II (n=344, 50%), Stanford type III (n=219, 32%), Stanford I (n=58, 9%) followed by Stanford IV (n=57, 8%) which equates to life-threatening symptoms according to Kishi classification (grade III and IV). The most common stent diameter was 12 mm (IQR: 10–16 mm) and the median length of the lesion was 6 cm (IQR: 3–14 cm). The median length of follow up was 54 days (IQR: 1–1849 days) with mean of 331 days. The median time to complete symptom resolution was 2 days (IQR: 0–5 days). There was an average of 1.5 stents per case in the entire series. The 30-day complication incidence was (n=62, 8%). This ranged from stent migration, malposition, failure to deploy, collapse (radial force) and immediate thrombosis. The 30-day mortality from the procedure was (n=21, 3%) from pericardial effusion, heart failure and rupture (**Table 2**).

**Table 2:** Table 2 The overall information of all the cases that were subjected to Wallstent for SVC syndrome

Category	Outcome
Total cases	n=701
Total stents	n=930
Malignancy	n=642 (92%)
Benign	n=36 (5%)
Male	n=548 (78%)
Female	n=152 (22%)
Age (median)	60 years (IQR: 26–89)
Stanford Type I	n=58 (9%)
Stanford Type II	n=344 (50%)
Stanford Type III	n=218 (32%)
Stanford Type IV	n=57 (8%)
Median lesion length	6 cm
Stents per case (average)	1.5 stents
Common stent diameter	12 mm (IQR: 10–16 mm)
Time to resolution	2 days (IQR: 0–5 days)
Follow up (median)	54 days (IQR: 1–1849 days)
30-day mortality	n=21 (3%)
30-day complications	n=62 (8%)
Immediate thrombosis	n=22 (35%)
Premature thrombosis	n=20 (32%)
Stent malposition	n=10 (16%)
Failure to deploy	n=5 (8%)
Migration	n=5 (8%)
Malignant cases	
30-day complication	n=53/643 (8.2%)
30-day mortality	n=20/643 (3%)
Non-malignant cases	
30-day complication	n=9/36 (25%)
30-day mortality	n=1/36 (2.7%)

SVC: superior vena cava; IQR: interquartile range

### Malignant versus non malignant

Overall mortality (n=21, 3%) and complication was (n=62, 8%) for both groups.

Amongst n=36 treated non-malignant cases, mortality was 2.7% (n=1/36) and complication incidence was 25% (n=9/36). Amongst n=643 treated malignant cases, mortality was 3% (n=20/642) and complication incidence was 8.2% (n=53/643).

The cumulative median patency of Wallstent for non-malignant aetiology was 550 days (IQR: 14–1080 days) versus malignant ones which was 120 days (IQR: 0–925 days).

### Sub-group analysis

**Figure 2:**
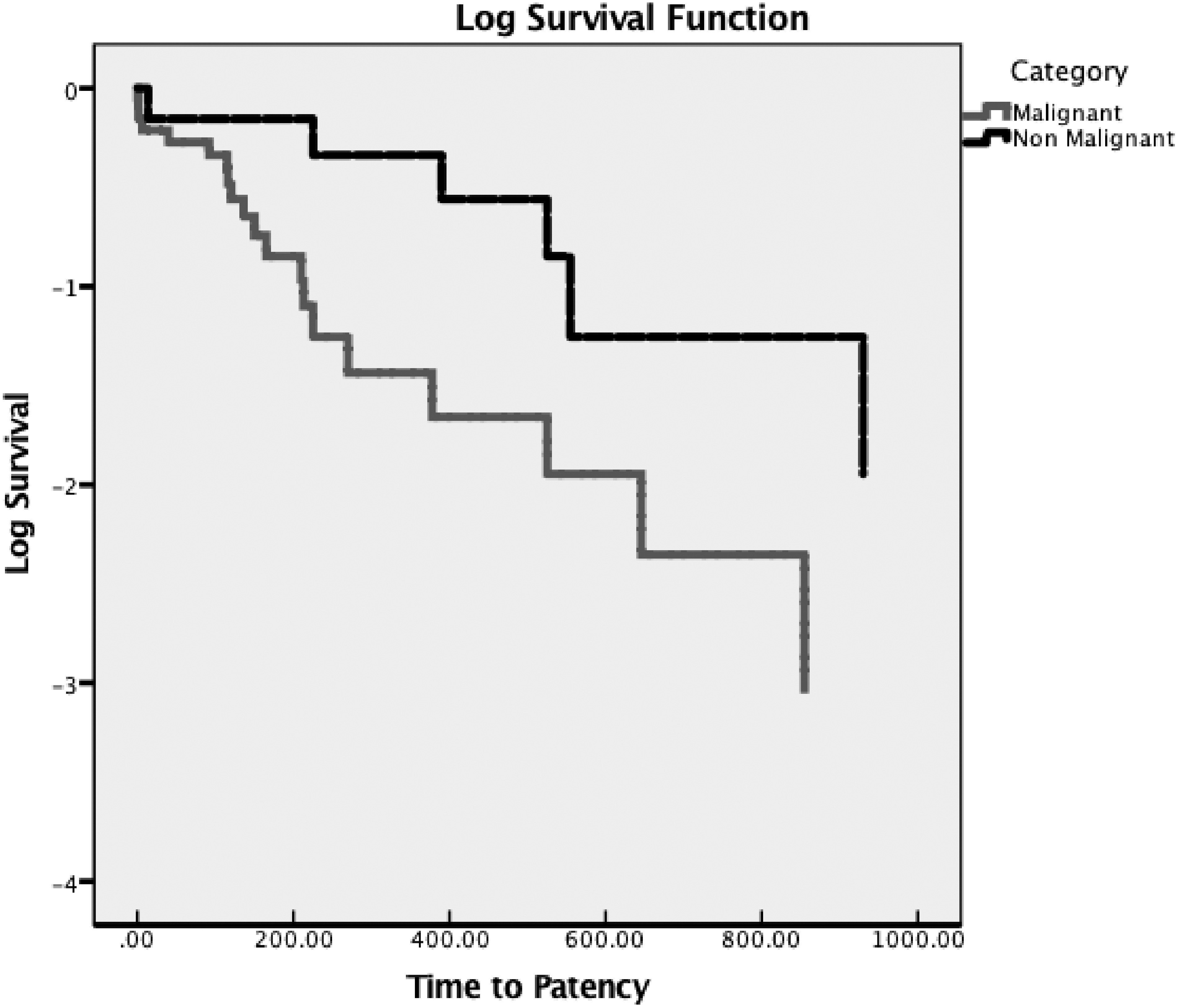
Fig. 2 Cumulative patency of the Wallstent in non-malignant vs. malignant case was [550 days (IQR: 14–1080) vs. 120 days (IQR: 0–925)] Log Rank (<0.03).

Data was further analysed for identification of attributes that might influence the endpoint of 30-day mortality and morbidity. The test of statistics on the endpoint of 30-day morbidity amongst all attributes was significant only on female gender (<0.03) (**Table 3**). This evaluation on the endpoint of mortality (30-day) demonstrated that no attribute is statistically significant. The survival analysis (Kaplan–Meier) demonstrated longer patency of the Wallstent in non-malignant cases in comparison to malignant ones (<0.03) (**Fig. 2**). The cumulative median patency of Wallstent for non-malignant aetiology was 550 days (IQR: 14–1080 days) versus malignant ones which was 120 days (IQR: 0–925 days).

**Table 3:** Table 3 Binary evaluation of attributes (variables) on the endpoint of 30-day mortality

Variables	Significance (p value)
Female gender	p=0.03
Male gender	p>0.5
Median age	p>0.5
Stanford I	p>0.5
Stanford II	p>0.5
Stanford III / Kishi III	p>0.5
Stanford IV/ Kishi IV	p>0.5

IQR: interquartile range

## Discussion

There is currently no consensus on the management of SVC syndrome to conform an evidence-based practice. The traditional modality of treatment in malignant cases, has been chemotherapy, radiotherapy, open surgery and bypass. Open approach using spiral vein graft, allografts or prosthetic graft have been promising, however their longevity due to further compression or low flow state remains poor.^12,13)^ In addition, most patients due to poor physiological reserve or function could not tolerate an open sternotomy and such could result in early mortality. SVC syndrome is also associated with multiple benign aetiologies as a consequences of intrinsic and extrinsic sequel. Despite their treatment, majority of benign cases still proceed to SVC syndrome and required resolution.^1,8,14–20)^

In contemporary practice, endovascular therapy (ET) has gained significant attention as a first line option for the treatment of SVC syndrome as long as it does not preclude or impact the outcome of future open surgery.^21)^ This practice is not supported by robust evidence (systematic review or randomisation) and the use of Wallstent amongst other endo-prosthesis is of no exception.^22)^ The outcome of this review demonstrates that wallstent within 48 h as a first line option, could result in rapid and complete resolution of symptoms with relative low mortality (3%).^23–31)^ This is an important outcome when majority of treated cases were of malignant nature (92%) categorised to Stanford type II (n=344, 50%) and type III (n=219, 32%) and secondly associated with life-threatening symptoms according to Kishi classification (grade III and IV).^8,32–40)^ This review also showed that the type of venographic or symptomatic classification has no clinical implication on endpoint of mortality making it more desirable as a first choice of therapy.

In this review, a total of sixty-two cases (8%) had complications within the 30-days of procedure with no reports of stent fracture. This ranged from immediate stenosis (n=22, 35%), premature thrombosis (n=20, 32%), stent malposition (n=10, 16%), failure to deploy (n=5, 8%) and migration (n=5, 8%). Immediate thrombosis was overcome by successful percutaneous thrombolysis in all cases and stent re-stenosis with in-stent successful venoplasty. Migration retrieval was achieved in three cases (n=3), with one resulting in mortality and other with oversize stent placement. It is worth mentioning that Wallstent has weaker edges than its main body and its deployment within disease segment or under extrinsic compression makes it more susceptible to early collapse and premature thrombosis. In addition, this is a braided stent (matrix design) and its deployment lacks detailed accuracy making it technically challenging with short landing zones. Therefore, complications such as stent collapse, premature stenosis and migration are inevitable but should remain minimal specially in the female cohort where this is notable (**Table 3**). The reason behind this attribute is unclear and could be a type II error.

Another technical aspect which is operator dependent and subjected to open debate is, unilateral or bilateral (kissing) stent and so called “Y” stent placement. This modes operandi, is attributed to SVC diameter of more than 15 mm and concomitant bilateral brachiocephalic vein involvement.^25)^ Amongst all retrieved articles, a complete comparative analytics was only available in one study that demonstrated lower complications in unilateral stent placement (p<0.03) with better longevity.^25)^ However, such practice continues to be case dependent and results are variable in practice.^41–43)^

The main objective of Wallstent stent placement in malignant SVC syndrome, is longevity (patency) prior to patient secondment to death due to their malignancy. The median primary patency in malignant cohort was 120 days (IQR: 0–925) (4-months) (mean of 7.1 months) which is arguably an acceptable patency for palliative group of patients. In contrast, the primary patency in non-malignant group was clinically and statically longer [550 days (IQR: 14–1080) (18-months)]. This raises the clinical question as to whether open surgical repair instead of Wallstent in later cohort could possess a better longevity.^44)^ This debate demands randomisation or comparative analysis which is not within the merit of this review however, it advocates further investigation.^44)^ The details of secondary patency as a subsequence of stent thrombolysis and re-plasty was not detailed for an objective inference thus no conclusion could be drawn.

The role of anticoagulation or antiplatelet was not meticulously reported within the retrieved articles prior or following the Wallstent placement specifically in malignant cohort. There is currently no consensus on the dose or indication of the aforementioned therapies in malignant SVC syndrome.^45,46)^ The lack of consensus is perhaps originated from the argument that angiogenesis within tumour could potentially result in procedural bleed and further re-intervention (thrombolysis or venplasty) could possibly be contraindicated.^47)^ Finally, studies to date have failed to confer any benefit for the prophylactic or treatment dose of anticoagulation or antiplatelet in practice.^48)^ In two studies, the use of antiplatelet did not demonstrate any benefit in terms of primary or secondary patency and finally in the study of Razton et al. the use of anticoagulation was associated with lower incidences of stent occlusion (hazard ratio 0.47, 95% confidence interval 0.2–1.13) with no statistical significance.^18,19,48,49)^

### Limitations

The standard of reporting within the retrieved articles lacked conformity. Amongst them, the details of secondary patency, symptom presentation, anticoagulation and antiplatelet were not available. In addition, the terminology of technical success and primary patency was commonly interchanged. In some series, it was not clear as the higher number of stents were due to technical failure, longer lesions or whether this was unilateral or bilateral stenting technique. Overall a meta-analysis would have been more optimal for the external validity, however, lack of comparative dataset created this limitation.

## Conclusion

It appears that the use of Wallstent as a first line approach with median patency of 120 days, mortality of 3% and complications of 8% amongst other stents in the treatment of malignancy induced SVC syndrome might be justified. This might be an acceptable approach where an open intervention due to palliative nature of the malignancy is not feasible. Their use in benign cohort, demonstrates a longer patency (550 days) but higher associated complications (25%). Therefore, the question arises as to whether open procedure could be an alternative and a comparative analysis might be advocated (stented non-malignant versus open surgery). The standard of reporting for endovascular therapy in SVC syndrome demands robust and universal definitions to achieve an objective clinical inference specially for benign cases.
